# Criteria for Treatment Response in Myasthenia Gravis: Comparison Between Absolute Change and Improvement Percentage in Severity Scores

**DOI:** 10.3389/fneur.2022.880040

**Published:** 2022-06-02

**Authors:** Hong-Yan Li, Ping Jiang, Yanchen Xie, Bing Liang, Ling Li, Cuiping Zhao, Yao-Xian Yue, Hai-Feng Li

**Affiliations:** ^1^Department of Neurology, Qilu Hospital (Qingdao), Cheeloo College of Medicine, Shandong University, Qingdao, China; ^2^Department of Neurology, Xuanwu Hospital, Capital Medical University, Beijing, China; ^3^Department of Neurology, Beijing Friendship Hospital, Capital Medical University, Beijing, China

**Keywords:** myasthenia gravis, criteria, treatment response, improvement percentage, severity

## Abstract

**Background:**

The absolute change in the severity score between the baseline and pre-specified time frame (absolute criterion) was recommended as a criterion for myasthenia gravis (MG) treatment response. But heterogeneity of disease severity might dilute major changes in individual patients. The rationality of relative criterion (improvement percentage) had not been evaluated in treatment response in patients with MG.

**Objectives:**

To investigate the consistency between an absolute criterion and a relative criterion in the evaluation of treatment response in patients with MG.

**Methods:**

We retrospectively analyzed the treatment response to a 3-month standardized treatment protocol with only glucocorticoid in 257 MG patients native to immunological treatments. With the commonly used absolute criterion, cut-offs of relative criteria were generated with the receiver operating characteristic (ROC) curve in the whole cohort and in patients with different degrees of baseline severity stratified by pre-treatment quantitative myasthenia gravis score (QMGS). The consistency between absolute and relative criteria was examined with Cohen's Kappa test and Venn diagrams.

**Results:**

The absolute and relative criteria had an overall substantial consistency (Kappa value, 0.639, *p* < 0.001) in the cohort. The Kappa values were substantial to almost perfect in mild and moderate groups and moderate in severe groups between the absolute and relative criteria (all *p* ≤ 0.001). More patients were classified as responsive with an absolute criterion while as unresponsive with a relative criterion in the moderate and severe groups.

**Conclusions:**

The overall consistency between absolute and relative criteria was substantial in the whole cohort. The inconsistency between the two criteria was mainly from the moderate or severe patients at the baseline.

## Introduction

In the guideline for clinical trials of myasthenia gravis (MG), quantitative measure, such as the MG composite, was recommended for determining improvement and worsening for patients with MG. Other quantitative measures were encouraged to be validated for the same purpose. The absolute change in the severity score between the baseline and pre-specified time frame was recommended as the criterion for treatment response (1). The quantitative myasthenia gravis score (QMGS) is a validated and frequently used measure in clinical trials and observational studies. Barohn et al. reported the interrater reliability of QMGS and considered the change of QMGS of > 2.6 points as clinical significance (2). In a study that assessed the responsiveness of QMGS, Bedlack (3) reported an average decrease of 2.3 points in the improved group. Minimal difference has been established for clinical trials of MG, which showed a QMGS change cut-off ≤ 3, was clinically important (4). However, the difference derived from group comparison is unfeasible when used in defining the responsiveness of individual patients to a given treatment. In a genetics study of glucocorticoid (GC) sensitivity, Xie et al. (5) used the definition of “improvement ≥ 3 points in QMGS or QMGS decreased to 0 after a 3-month GC treatment” as the criterion to analyze the factors that might be associated with the short-term sensitivity to GC.

The heterogeneity of disease severity might dilute major changes in individual patients by comparison at the group level, particularly in patients with mild and severe involvement. In our correspondence to this guideline (6), we proposed using a relative score that is based on the improvement percentage of an individual patient during the interval for treatment response evaluation. The relative score was defined as (score_pre–*treatment*_ − score_post–*treatment*_)/score_pre–*treatment*_. In China, such a relative scoring system had been used for more than 25 years (7). The relative score may provide a useful individualized evaluation of therapeutic effects and can be analyzed as a linear parameter. Furthermore, comparison of the proportions of patients in both treatment and placebo groups who met a pre-specified effect criterion based on the relative score may provide us with another view of the treatment effects, even if between-group comparisons showed no significant differences. In a genetic study on rheumatoid arthritis, in which definition of individual treatment effect was essential, a similar criterion based on improvement percentage was used (8). In reply to our correspondence (6, 9), the authors stated that skewed distributed baseline severity and relevant stratification of disease severity might lead to potential bias in using a relative score as a criterion.

Glucocorticoids are the first-line immunosuppressive treatment for MG because of their rapid effect and controllable side effects (10, 11). Large-size retrospective studies have shown significant improvement in patients with MG with different doses of GCs. The mean duration between the onset to improvement after GC treatment was 13~14 days; the mean onset to sustained improvement was 1.5~3 months (12). Hence, the responsiveness to GCs is a good example of a short-term treatment effect.

In this study, we retrospectively analyzed the treatment response in patients with MG treated with a standardized 3-month protocol with only GCs and compared the criterion based on absolute change of QMGS and percentage of QMGS improvement after the treatment. Due to the skewed distribution of the pre-treatment QMGS in this study, we stratified them into mild, moderate, and severe subgroups to explore the influence of baseline QMGS on the consistency of the criteria.

## Materials and Methods

### Patient Recruitment and Study Design

A total of 257 patients with MG, who had not received any immunological treatments were consecutively enrolled and followed every month till 3 months after treatment, were included in this study. After the pre-treatment QMGS were recorded, GCs equivalent to 0.75 ~ 1 mg/kg/day of prednisone were started. The dosage of GCs was tapered gradually when there was notable improvement, or remained the same as the initial dosage until the end of 3 months. The post-treatment QMGS were recorded. Details of patient recruitment and treatment were expatiated in our previous research (5).

Criterion A was set based on the change of QMGS (QMGS_pre−treatment_ − QMGS_post–*treatment*_). Improvement ≥ 3 points in QMGS or QMGS decreased to 0 after 3-month treatment was defined as responsive to GCs (2, 3). Criterion R was set based on improvement percentage as (QMGS_pre–*treatment*_ − QMGS_post−treatment_)/QMGS_pre–*treatment*_. Taking the criterion A as the reference standard, we used receiver operating characteristic (ROC) curve to define the optimum cut-offs for the criterion R in the whole group and three subgroups stratified by pre-treatment QMGS. The consistency was compared between the two criteria in the whole group, as well as in subgroups.

### Statistical Analysis

Statistical analyses were performed using IBM SPSS version 20.0 (SPSS Inc., Chicago, IL, USA). The normality of continuous variables was tested by the Kolmogorov–Smirnov test. Continuous variables were presented as mean ± standard deviation (SD) or median (interquartile range, IQR). Categorical variables were expressed as frequencies (percentages), and the chi-square or Fisher's exact test was used to compare their differences. The optimum cut-offs of criterion R for GC repressiveness were determined by ROC curves (13). Two × two tables were constructed for GC responsiveness based on relevant cut-offs. Cohen's Kappa test was used to analyze the consistency between the two criteria. Kappa values of 0.21~0.4 were considered fair, 0.41~0.60 moderate, 0.61~0.80 substantial, and 0.81~1.00 almost perfect (14). A two-tailed *p* < 0.05 was considered significant. Venn diagrams were used to demonstrate the consistent and inconsistent patients by the two criteria, and details of improvement of the inconsistent patients were listed for inspection.

## Results

### General Characteristics

**Figure 1:**
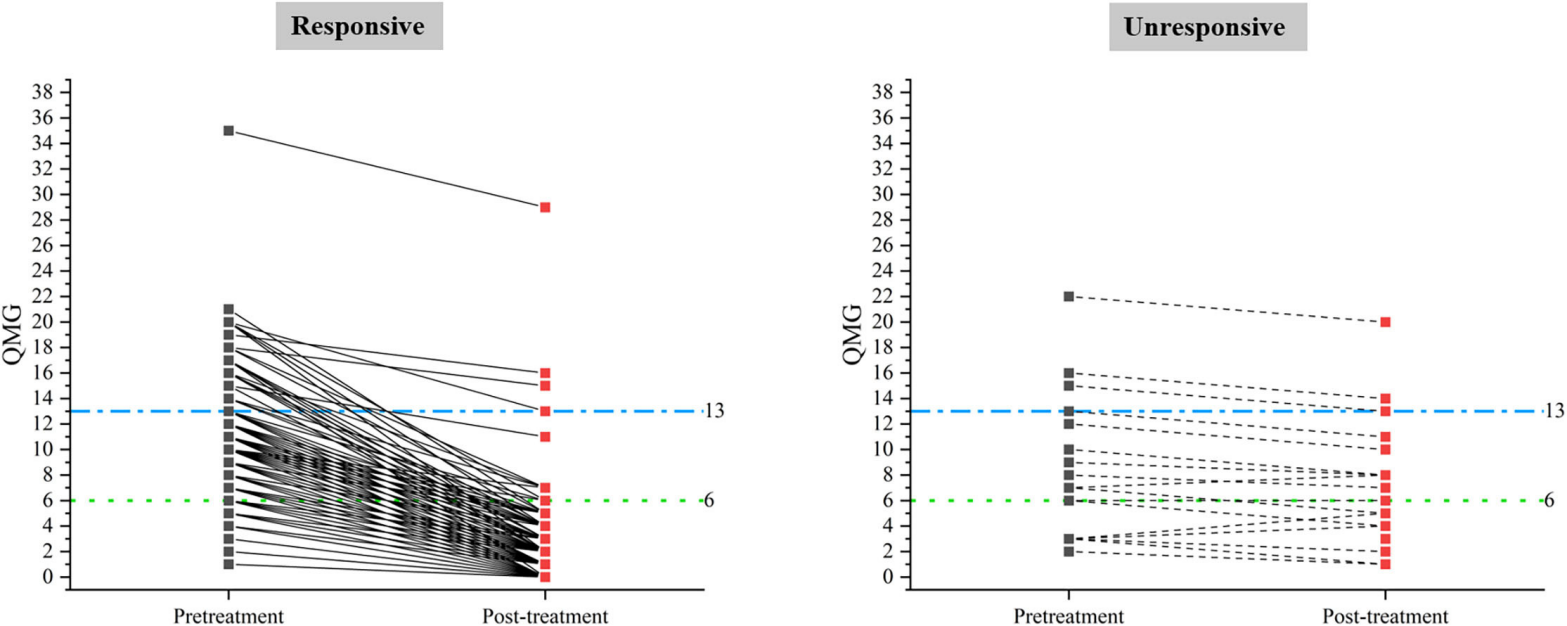
Pre-treatment and post-treatment QMGS in responsive and unresponsive patients classified by criterion A.

A total of 98 (38.1%) male patients and 159 (61.9%) female patients were included in this study. Onset age ranged from 15 to 80 years old (43.4 ± 16.6). The disease duration prior to treatment ranged from 2 to 48 months (median 4, IQR 2 ~ 11). The pre-treatment QMGS ranged from 1 to 35 (median 6, IQR 4 ~ 11). The patients were classified into three subgroups according to baseline QMGS as follows: 105, mild (QMGS 1 ~ 5); 108, moderate (QMGS 6 ~ 12); and 44, severe (QMGs ≥ 13) patients. After 3-month GC treatment, the change of QMGS ranged from −2 to 18 (median, 5; IQR, 3 ~ 8). The demographic and clinical features were summarized in Supplementary Table 1, and the changes in absolute QMGS were shown in Figure 1.

### Responsiveness to GCs

The absolute QMGS changes ranged from −2 to 18 (median, 5; IQR, 3 ~ 8). The improvement percentages ranged from −66.7 to 100% (median, 86.67%; IQR, 70 ~ 100%). Based on criterion A, 235 patients (91.44%) were classified as responsive to GCs, and 22 patients (8.56%) as unresponsive. There were significant differences in absolute changes of QMGS (*p* < 0.001) and improvement percentage of QMGS (*p* < 0.001) between responsive and unresponsive groups. There was a significant difference in disease duration before GCs treatment (≤ 6 months vs. > 6 months, *p* = 0.027) between the two groups. No differences were found in other clinical characteristics between the two groups (Supplementary Table 1).

Using the ROC method, an improvement of 51.925% was calculated as the optimum cut-off for criterion R in the whole group. The cut-offs were calculated as 70.835, 36.665, and 15.585% in the mild, moderate, and severe subgroups, respectively (Supplementary Table 2).

### Consistency Between Criterion A and Criterion R

Using the cut-off (51.925%, Criterion R1) derived from all the patients, the Kappa value was 0.639 in the whole group, 0.824 in the mild group, 0.639 in the moderate group, and 0.462 in the severe group (all *p* ≤ 0.001, Table 1). Because the proportion of patients classified into the moderate group by Criterion A was the largest among the three subgroups, and moderate baseline QMGS was often seen in clinical trials, we used the cut-off derived from these patients (36.665%) to set Criterion R2. With criterion R2, the Kappa values were 0.735, 0.713, 0.826, and 0.56 in the whole group, mild group, moderate group, and the severe group, respectively (all *p* ≤ 0.001, Table 1). The Kappa values were substantial to almost perfect in the mild and moderate groups and moderate in the severe group between Criterion A and both Criteria R1 and R2.

**Table 1:** Consistency analysis between Criterion A and Criterion R.

	**A+/R+**	**A+/R-**	**A-/R-**	**A-/R+**	**Kappa value**	***P*-value**
**Criterion R1 (cut-off at 51.925%)**						
Total	218	17	20	2	0.639	<0.001
1–5	98	0	5	2	0.824	<0.001
6–12	87	10	11	0	0.639	<0.001
≥13	33	7	4	0	0.462	0.001
**Criterion R2 (cut-off at 36.665%)**						
Total	226	9	19	3	0.735	<0.001
1–5	98	0	4	3	0.713	<0.001
6–12	93	4	11	0	0.826	<0.001
≥13	35	5	4	0	0.56	0.001

**Figure 2:**
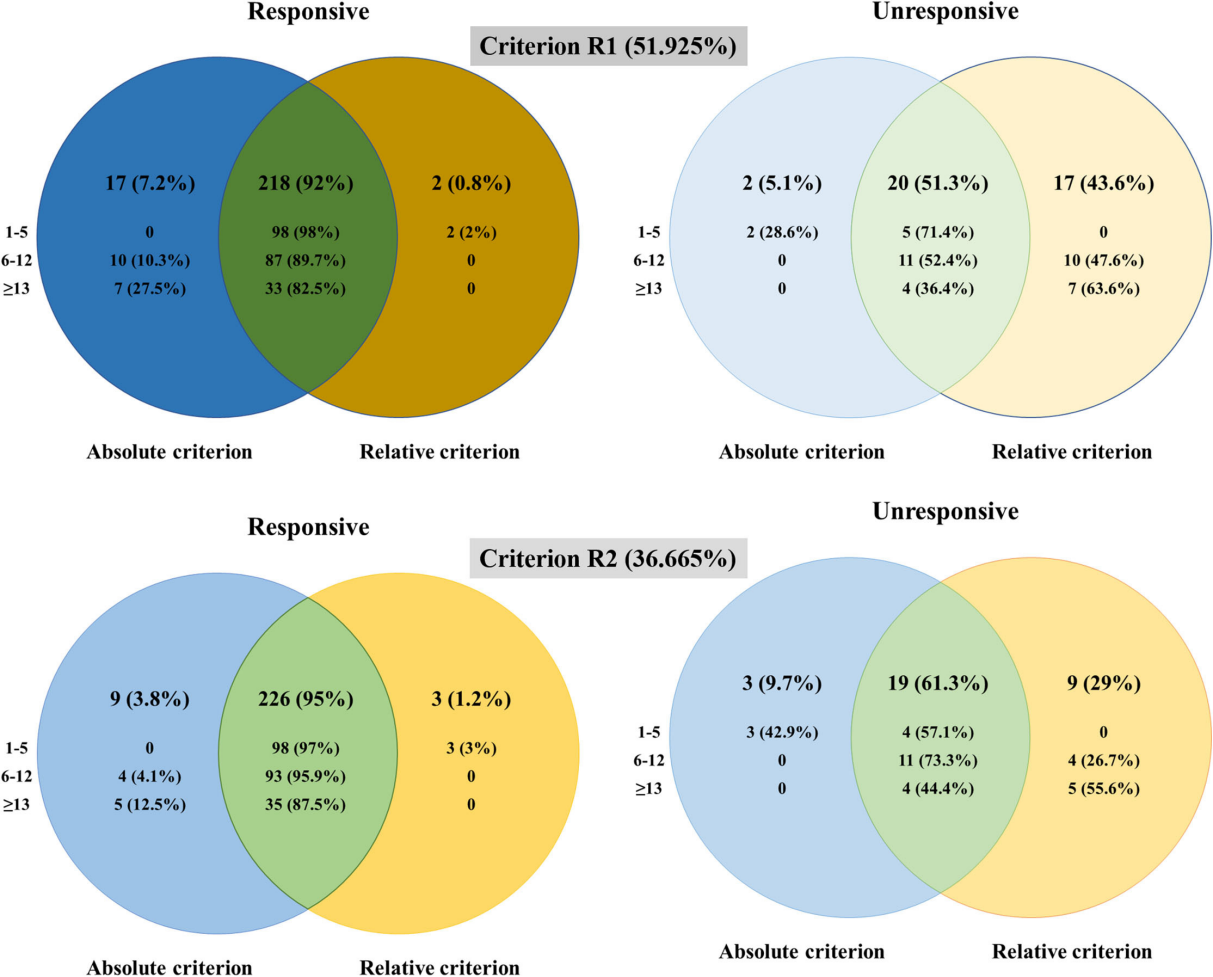
The differences in the patients classified as responsive and unresponsive with different criteria.

The Venn diagrams (Figure 2) demonstrated that two patients were classified as unresponsive with Criterion A while as responsive with Criterion R1, three patients (including the above two patients) as unresponsive with Criterion A, while as responsive with Criterion R2. This inconsistent pattern was only seen in the mild group. The proportions of patients classified as responsive in the mild group were 98/105 (Criterion A), 100/105 (Criterion R1), and 101/105 (Criterion R2), indicating a strong consistency between Criterion A and Criterion R in the mild group. Even though the changes of QMGS did not reach 3 points, the improvement percentages were 50~66.7% in these three patients. Seventeen patients were classified as responsive with Criterion A while unresponsive with Criterion R1, and nine patients (included in the above 17 patients) were classified as responsive with Criterion A while unresponsive with Criterion R2. This inconsistent pattern was only seen in the moderate and severe groups. The proportions of the patients classified as unresponsive in the moderate group were 11/108 (Criterion A), 21/108 (Criterion R1), and 15/108 (Criterion R2); unresponsive in the severe group were 4/44 (Criterion A), 11/44 (Criterion R1), and 9/44 (Criterion R2). Even though the change of QMGS reached 3 points, the improvement percentages were 15.79~50% in the unresponsive patients defined with Criterion R1 and 15.79~35% in the unresponsive patients defined with Criterion R2 (Table 2).

**Table 2:** Clinical features of inconsistent patients in Criterion A and Criterion R.

**Criterion**			**Gender**	**Onset age**	**Thymoma**	**AChRAb**	**MuSKAb**	**Onset involvement**	**Treat in 6 months**	**QMGS-pre**	**QMGS-post**	**Δ QMGS**	**Δ %**
**A**	**R1**	**R2**											
-	-	+	Male	17	-	-	-	Ocular	-	2	1	1	50
-	+	+	Male	68	-	+	-	Ocular	-	3	1	2	66.67
-	+	+	Female	60	-	-	-	Ocular	-	3	1	2	66.67
+	-	-	Male	42	+	+	-	Ocular	-	19	16	3	15.79
+	-	-	Male	34	+	+	-	Generalized	+	18	15	3	16.67
+	-	-	Male	46	+	+	-	Ocular	+	35	29	6	17.14
+	-	-	Female	59	-	+	-	Generalized	+	15	11	4	26.67
+	-	-	Female	46	-	-	-	Generalized	-	10	7	3	30
+	-	-	Male	25	-	+	-	Generalized	+	10	7	3	30
+	-	-	Female	54	-	+	-	Generalized	+	10	7	3	30
+	-	-	Female	20	-	+	-	Generalized	+	10	7	3	30
+	-	-	Female	46	+	+	-	Ocular	+	20	13	7	35
+	-	+	Female	32	+	+	-	Ocular	-	10	6	4	40
+	-	+	Male	75	+	+	-	Generalized	+	10	6	4	40
+	-	+	Female	42	+	+	-	Ocular	+	9	5	4	44.44
+	-	+	Female	72	-	-	+	Ocular	+	6	3	3	50
+	-	+	Female	54	+	+	-	Generalized	-	8	4	4	50
+	-	+	Female	31	-	+	-	Ocular	+	10	5	5	50
+	-	+	Female	36	+	+	-	Ocular	-	14	7	7	50
+	-	+	Female	59	+	+	-	Ocular	-	14	7	7	50

## Discussion

A recent study that reported the change in % of normal between original and follow-up visits has shown a strong correlation with the change in QMGS (ΔQMGS) (15), which suggested the potential usage of improvement percentage as the response criterion. In our study, the consistencies were substantial between criteria (A vs. R1 and A vs. R2) in all the patients, substantial to almost perfect in the mild and moderate patients while moderate in the severe patients. The Venn diagrams confirmed the inconsistency came from baseline moderate and severe patients.

The two criteria were developed at the group level or the individual level. The confounding role of baseline severity on responsiveness in an individual patient was also noted by Katzberg et al. (4). They proposed using a QMGS cut-off of 2 for patients with a baseline QMGS of <16 and 3 for those with baseline QMGS > 16. In our study, we used different cut-offs to set Criteria R1 and R2, which resulted in a different consistency. However, the improvement percentages in individual patients were the same whichever the criterion R was used. From the detailed information on inconsistent patients, the diluting effects of baseline severity on responsiveness could be visualized directly. When two patients with the same ΔQMGS of 4 were taken as an example, QMGS decreased from 15 to 11 in one patient, while from 8 to 4 in the other patient. In baseline moderate or severe patients with MG, using the improvement percentage of 36.665% (Criterion R2) as the cutoff of QMGS is closer to our clinical experience.

There were several limitations in our study: First, the pre-treatment QMG score in this study was in skewed distribution; the number of severe patients was much less than the mild and moderate ones. However, skewed data were inevitable in clinical studies. We used the cut-off derived from moderate patients, which constituted the largest proportion of all the patients to overcome this limitation, and acquired substantial consistency between the absolute and relative criteria. However, comparison at the group level could not overcome the bias from skewed distribution in baseline QMGS. The patients who had high baseline scores but smaller Δ QMGS might not have actual improvements, as shown in our study. Second, we lack another reference criterion for which the two criteria could be compared, especially simple patient-reported measures, such as single simple questions (15) or scales, such as MG-ADL or MG-QOL15. Nevertheless, in the short-term evaluation with an interval of 3 months, the slope of the connecting line (pre-treatment QMGS to post-treatment QMGS) in an individual patient might give a clue for the evaluation of the treatment effect. The larger the slope is, the stronger the response is.

## Conclusion

By determination of the consistency between absolute and relative criteria, this study showed an overall substantial consistency in the short-term treatment response of GC in patients with MG and the inconsistent aspects between the two criteria in subgroups stratified by baseline severity. This will shed light on the definition of responsiveness in both observational studies and clinical trials in MG. The relative criterion should be examined with other quantitative measures of severity to define treatment response in patients with MG.

## Data Availability Statement

The original contributions presented in the study are included in the article/Supplementary Material, further inquiries can be directed to the corresponding author/s.

## Ethics Statement

The studies involving human participants were reviewed and approved by Ethics Committee of Beijing Friendship Hospital, Capital Medical University and Ethics Committee of Affiliated Hospital of Qingdao University. Written informed consent to participate in this study was provided by the participants' legal guardian/next of kin.

## Author Contributions

H-FL and Y-XY conceptualized and designed the study and revised the manuscript. H-YL and PJ interpreted the data and wrote the manuscript. H-FL, YX, and Y-XY diagnosed, treated, recruited, and followed up with the patients in this study. H-YL, PJ, and BL performed the statistical analysis. LL and CZ contributed to the discussion. All authors contributed to the article and approved the submitted version.

## Funding

This study was supported by the National Natural Science Foundation of China (Nos. 81070963, 81771362, and 82171397 to H-FL), the Qingdao Technology Program for Health and Welfare (No. 17-3-3-26-nsh to H-FL), and Research Grant from Qilu Hospital (Qingdao), Cheeloo College of Medicine, Shandong University (QDKY2021RX06 to Y-XY).

## Conflict of Interest

The authors declare that the research was conducted in the absence of any commercial or financial relationships that could be construed as a potential conflict of interest.

## Publisher's Note

All claims expressed in this article are solely those of the authors and do not necessarily represent those of their affiliated organizations, or those of the publisher, the editors and the reviewers. Any product that may be evaluated in this article, or claim that may be made by its manufacturer, is not guaranteed or endorsed by the publisher.
