# Supplementary material for: Criteria for Treatment Response in Myasthenia Gravis: Comparison Between Absolute Change and Improvement Percentage in Severity Scores
Source: Front Neurol. 2022 Jun 2;13:880040. doi: 10.3389/fneur.2022.880040 (PMC9201395; doi:10.3389/fneur.2022.880040)
Supplement: Supplementary file 1 [file Table_1.docx]

Suppl Table 1 General characteristics in patients classified with absolute criterion

| Groups | Responsive  (n=235) | Unresponsive  (n=22) | P value |
| --- | --- | --- | --- |
| Patient characteristics | | | |
| Gender |  |  |  |
| Male | 89 | 9 | 0.780 |
| Female | 146 | 13 |  |
| Onset age |  |  |  |
| < 50 years | 152 | 13 | 0.601 |
| ≥50 years | 83 | 9 |  |
| Onset involvement |  |  |  |
| Ocular | 163 | 14 | 0.584 |
| Generalized | 72 | 8 |  |
| Thymoma |  |  |  |
| Positive | 56 | 7 | 0.417 |
| Negative | 179 | 15 |  |
| AChR-Ab |  |  |  |
| Positive | 163 | 16 | 0.686 |
| Negative | 63 | 5 |  |
| MuSK-Ab^a^ |  |  |  |
| Positive | 7 | 1 | 0.476 |
| Negative | 56 | 4 |  |
| Disease duration before steroids |  |  |  |
| ≤ 6 months | 163 | 10 | 0.027^b^ |
| > 6 months | 72 | 12 |  |
| Absolute and relative changes of QMGS | | | |
| Absolute changes | 5 (3, 8) | 2 (0.75, 2) | <0.001^c^ |
| Improvement percentage (%) | 90 (75, 100) | 16.03 (6.82, 33.33) | <0.001^d^ |

1. MuSK-Ab was detected in AChR (-) patients with radioimmunoassay kit
2. There was significant difference in disease duration before steroids (≤ 6 months vs. > 6 months) between responsive and unresponsive groups
3. There was significant difference in absolute changes of QMGS between responsive and unresponsive groups
4. There was significant difference in improvement percentage of QMGS between responsive and unresponsive groups

Suppl Table 2 Cut-offs of relative criterion in the whole group and subgroups

|  | Cut-offs | AUC | 95%CI | P value | Sensitivity | Specificity | Youden index |  |
| --- | --- | --- | --- | --- | --- | --- | --- | --- |
| Total | 51.925% | 0.975 | 0.953-0.996 | <0.001 | 92.8% | 90.9% | 0.837 |  |
| 1-5 | 70.835% | 0.985 | 0.963-1 | <0.001 | 94.9% | 100% | 0.949 |  |
| 6-12 | 36.665% | 0.993 | 0.98-1 | <0.001 | 95.9% | 100% | 0.959 |  |
| ≥13 | 15.585% | 1 | 1-1 | 0.001 | 100% | 100% | 1 |  |
